# Manifestaciones clínicas y evolución a largo plazo de tres casos de rosácea ocular atendidos en un hospital de alta especialidad del sureste de México

**DOI:** 10.7705/biomedica.5001

**Published:** 2020-06-30

**Authors:** Fiona Xacur-García, Rodrigo Díaz-Novelo, Linette Herrera-David, Paulina Moreno-Arjona, Nina Méndez-Domínguez

**Affiliations:** 1 Servicio de Oftalmología, Hospital Regional de Alta Especialidad de la Península de Yucatán, Mérida, Yucatán, México Hospital Regional de Alta Especialidad de la Península de Yucatán Yucatán México; 2 Escuela de Medicina, Universidad Marista de Mérida, Mérida, Yucatán, México Universidad Marista de Mérida Universidad Marista de Mérida Yucatán Mexico

**Keywords:** rosácea, oftalmopatías, técnicas de diagnóstico oftalmológico, pronóstico, trasplante, Rosacea, eye diseases, diagnostic techniques, ophthalmological, prognosis, transplantation

## Abstract

La rosácea es una alteración cutánea crónica que se ha asociado con factores etiológicos inespecíficos y diversas manifestaciones sistémicas. La rosácea cutánea suele evolucionar a rosácea ocular del 6 al 72 % de los pacientes. Al no existir criterios específicos que la caractericen, la rosácea ocular implica un reto diagnóstico. Para fortalecer la sospecha diagnóstica temprana, se presentan tres casos de pacientes con evolución clínica distinta, pero que tuvieron en común el retraso diagnóstico, lo que se tradujo en manifestaciones graves y daño ocular extenso.

## Abstract (en)

Rosacea is a chronic skin disorder that has been associated with unspecific etiological factors and diverse systemic manifestations. Cutaneous rosacea usually evolves to ocular rosacea in 6 to 72% of patients. In the absence of specific criteria to characterize this pathology, ocular rosacea can mean a diagnostic challenge. To strengthen early diagnostic suspicion, we present three cases of patients with different clinical evolution who had in common diagnostic delay resulting in severe manifestations and extensive ocular damage.

La rosácea es una enfermedad cutánea crónica que afecta las glándulas sebáceas, especialmente en la región central de la cara, y se describe como una alteración inflamatoria con diversas manifestaciones sistémicas. Su gravedad clínica es variable, y se desconocen el mecanismo fisiopatológico y su etiología ^1^. A menudo se diagnostica erróneamente, lo que puede producir resultados desfavorables.

El cuadro clínico se caracteriza por la aparición de eritema persistente, pápulas, pústulas y telangiectasias de diversos grados y de distribución simétrica en las regiones frontal, geniana, nasal y mentoniana ^2^. Aunque se la considera una enfermedad de la piel, en su estudio comparativo Arman, *et al*., estiman que la incidencia de la afectación ocular varía entre el 6 y el 72 % ^1^^-^^5^.

Puede haber compromiso primario solamente en la piel o acompañarse de manifestaciones oculares simultáneas, las cuales incluyen linfedema periorbitario, eritema en el margen del párpado y telangiectasia, blefaritis y secreciones espesas en las glándulas de Meibomio, orzuelo y chalación, ojo seco, colonización bacteriana, erosión corneal, irrigación y adelgazamiento, episcleritis y escleritis. La principal afectación ocular de la rosácea es la inflamación de las glándulas de Meibomio, cuyos orificios se dilatan y obstruyen provocando disfunción ^1^.

Se ha reportado que la edad de presentación inicial fluctúa entre los 30 y los 50 años, con una prevalencia de hasta el 10 %, especialmente en caucásicos de origen celta o del norte de Europa ^3^. La rosácea afecta a más de 16 millones de americanos, aunque se ha descrito en todos los grupos étnicos, incluidos latinos, asiáticos y afroamericanos, quienes suelen presentar formas granulomatosas de la enfermedad. Se ha sugerido que la pigmentación de la piel puede dificultar la detección de los signos característicos, lo que contribuiría al subdiagnóstico en los pacientes de piel oscura ^4^^,^^5^.

Las mujeres son diagnosticadas más frecuentemente que los hombres y tienden a ser detectadas de manera más oportuna, lo que podría explicarse porque buscan atención médica antes que los hombres; por esta razón, ellos presentan una mayor incidencia de complicaciones de la enfermedad. La rosácea afecta a todos los grupos de edad, aunque es más común en la adultez. Se considera que la rosácea pediátrica es poco frecuente, lo que podría deberse a que no se reporta, pues los signos dermatológicos suelen estar ausentes o ser difíciles de detectar ^5^.

En el presente reporte de casos, se describen las características clínicas de la rosácea ocular y sus diversas presentaciones en tres casos, con el objetivo de fortalecer el diagnóstico temprano de la rosácea ocular, y mejorar el pronóstico y la calidad de vida de los pacientes.

## Caso 1

Se trata de un hombre de 47 años originario de Centroamérica, residenciado en el momento del diagnóstico en el sureste de México y previamente en los Estados Unidos, sin antecedentes de importancia más allá de su trabajo como empleado en granjas y sembradíos.

Fue referido a la unidad especializada en córnea por presentar opacidades corneales bilaterales crónicas, así como perforación ocular en el ojo derecho. Su condición había sido diagnosticada y tratada como de origen herpético por diferentes médicos durante varios años. El paciente refería dolor y sensación de cuerpo extraño de dos meses de evolución.

**Figura 1.**
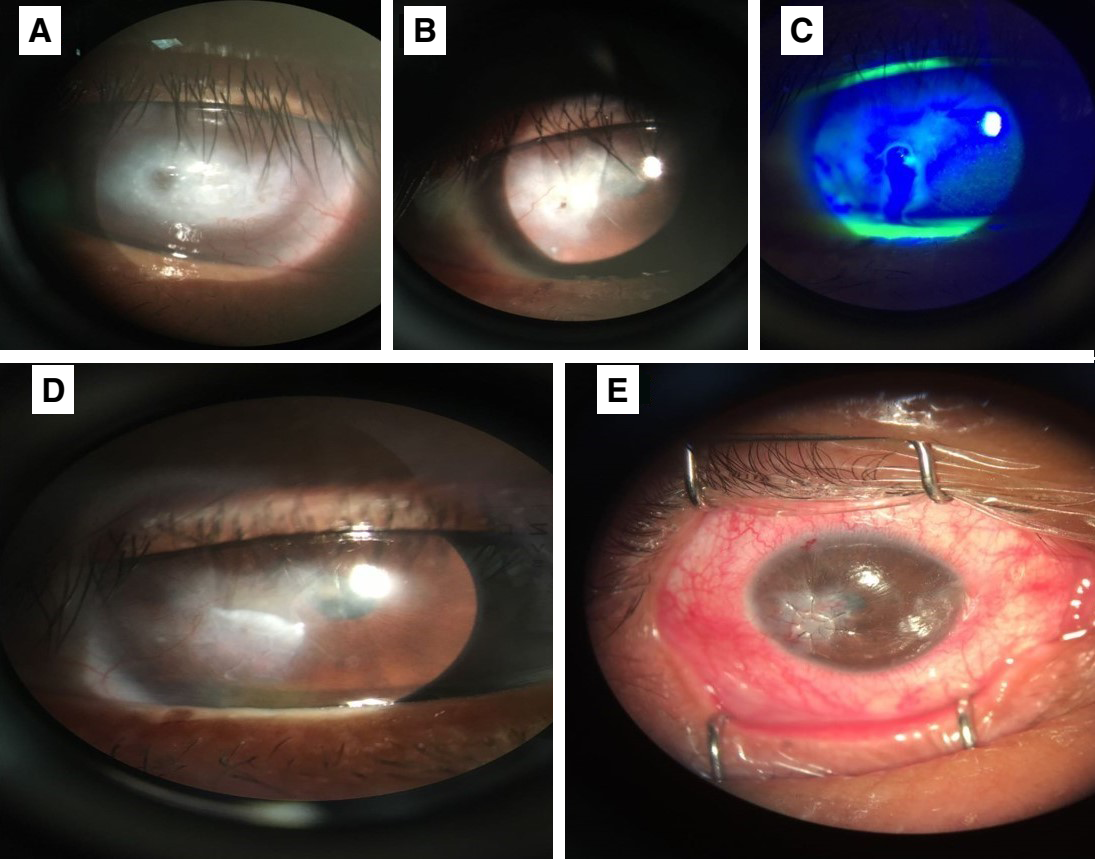
Imágenes correspondientes al caso 1 A) El ojo izquierdo con opacidad corneal densa del 70 % de la córnea que obstruye el eje visual y la irrigación en dos cuadrantes. B) Ojo derecho antes de la cirugía con opacidad e irrigación corneal en el sector infero-temporal y descematocele con microperforación corneal C) Ojo derecho antes de la cirugía con signo de Seidel espontáneo D) Ojo derecho a los cuatro meses de la cirugía. Se observa el parche corneal tectónico bien integrado, con irrigación y opacidad en el cuadrante infero-temporal que afecta parcialmente el eje visual, pupila normocórica, cámara anterior formada y cristalino claro. E) Vista del parche corneal tectónico en el ojo derecho inmediatamente después de la cirugía. Se observa el injerto tectónico que sella la perforación corneal.

En la exploración se encontró agudeza visual de 20/160 en el ojo derecho y percepción de movimiento de manos en el ojo izquierdo. En la biomicroscopía del ojo derecho, se evidenció opacidad corneal densa que afectaba parcialmente el eje visual, asociada con irrigación y compactación, presencia de descematocele con fuga acuosa (signo de Seidel) espontánea, cámara anterior formada y sinequias anteriores (figura 1). En el ojo izquierdo, la córnea presentaba opacidad central densa, bullas corneales, e irrigación superficial y profunda.

Al ser preguntado específicamente sobre la rosácea, el paciente confirmó que había sido diagnosticado con esta enfermedad cutánea ocho años atrás. Se diagnosticó perforación corneal secundaria a rosácea ocular y se lo programó para cirugía del parche tectónico en el ojo derecho, la cual se llevó a cabo sin complicaciones. Se le dio tratamiento con doxiciclina oral, sulfato de tobramicina y acetato de fluorometolona, así como un lubricante.

La agudeza visual después de la cirugía del ojo derecho fue de cuenta dedos. A los dos meses de la cirugía, se retiraron los puntos de sutura y, a los cuatro meses, se hizo una refracción lográndose una mejoría de la agudeza visual de 20/70 con +2,00=-3,50 x 57.

El paciente pasó a lista de espera para trasplante de córnea en el ojo izquierdo, con el fin de eliminar la opacidad y lograr la recuperación de la visión. El pronóstico del trasplante de córnea en este paciente era muy reservado, dada la naturaleza de la enfermedad, su diagnóstico tardío y la gravedad de la irrigación corneal extensa, que es factor de mal pronóstico para el trasplante.

## Caso 2

Se trata de un hombre de 37 años residente en la zona urbana que refirió disminución progresiva de la agudeza visual de larga evolución asociada por él con una lesión producida años atrás por una espina vegetal en el ojo izquierdo.

**Figura 2.**
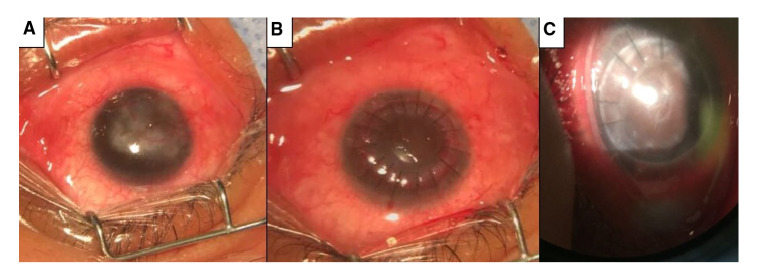
Imágenes correspondientes al caso 2 A) Ojo derecho antes de la cirugía. Se observa opacidad corneal central con irrigación en tres cuadrantes. No fue posible valorar los detalles del segmento anterior. B) Ojo derecho inmediatamente después de la cirugía. Se observa el injerto corneal *in situ* y restos del sangrado de los vasos corneales en interfase. C) Ojo derecho dos meses después de la cirugía. Se observa el injerto corneal *in situ* y opacidad central en interfase. En este momento ya se habían retirado algunos puntos de sutura.

En la exploración se encontró una agudeza visual de 20/20 en el ojo derecho y de 20/80 en el izquierdo; la presión ocular era de 14 mm Hg en ambos ojos (figura 2). En la biomicroscopía se encontró eritema y engrosamiento de los bordes palpebrales, con secreción de Meibomio espesa, hiperemia conjuntival, opacidades y vascularización corneal. Además, presentaba lesiones papulares y pústulas en la piel, por lo que se le diagnosticó rosácea con afectación ocular.

Se inició el tratamiento con doxiciclina, acetato de fluorometolona y sulfato de tobramicina (tópico), así como medidas de aseo, y se lo remitió al dermatólogo, quien únicamente le indicó evitar la luz solar.

A pesar del tratamiento médico, las opacidades corneales continuaron empeorando y la agudeza visual siguió disminuyendo. Al cabo de dos años, la agudeza visual del ojo derecho había disminuido hasta el nivel de percepción de movimiento de manos y, en el izquierdo, a 20/200, por lo que se le hizo un trasplante de córnea lamelar anterior con burbuja en el ojo derecho.

En la prueba de agudeza visual después de la cirugía, se observó una agudeza visual de 20/160 en el ojo derecho y, en el izquierdo, de 20/200. En la biomicroscopía se observó el botón corneal claro en el ojo derecho, estrías y opacidad localizada en la interfase que afectaba parcialmente el eje visual, neoangiogénesis (*vascularization*) profunda en M 10 a 12 y en M 4, cámara anterior formada, sin células, pupila normorrefléctica, cristalino claro y retina aplicada. En el ojo izquierdo, la córnea mostraba opacidades dispersas, que afectaban parcialmente el eje visual, y retina sin alteraciones.

El trasplante de córnea lamelar fue en el ojo derecho. Posteriormente, el paciente fue tratado con sulfato de tobramicina, prednisolona en solución oftálmica, tacrolimus y doxiciclina. Tres meses después del trasplante, hubo una disminución de la agudeza visual en el ojo operado y se encontró edema corneal sugestivo de rechazo del estroma, el cual se trató exitosamente con esteroides tópicos. El rechazo en los trasplantes lamelares anteriores es muy poco frecuente, sin embargo, el paciente con rosácea pierde el privilegio inmunológico de la córnea, por lo que es más propenso a esta complicación, y dado que la evolución no era la óptima, el pronóstico de la función visual es reservado.

## Caso 3

Se trata de una mujer de 70 años cuya enfermedad había comenzado ocho años atrás con sensación de cuerpo extraño, ojo rojo y disminución progresiva de la visión, que culminó en perforación en el ojo izquierdo. La paciente refirió haberse expuesto al sol y a altas temperaturas sin protección. Entre sus hábitos higiénicos y dietéticos, llamaron la atención el tabaquismo asociado con la dependencia a la nicotina y el etilismo periódico.

Ante el agravamiento de su cuadro clínico (disminución de la agudeza visual del ojo izquierdo, hiperemia, epífora, escozor, fotofobia y sensación de cuerpo extraño) acudió a consulta oftalmológica.

En la evaluación se observó una agudeza visual en el ojo derecho de percepción de movimiento de manos y en el izquierdo, de conteo de dedos a un metro. La presión ocular en el ojo derecho era de 18 mm Hg y, en el izquierdo, de 16 mm Hg. En la exploración llamó la atención la presencia de pupilas sin reflejos, con opacidad e irrigación corneal irregular, así como catarata. El fondo de ojo en el derecho no se pudo valorar por opacidad y, en el izquierdo, se halló una excavación de papila del 70 al 80 %. Se le hizo una tomografía de coherencia óptica y campo visual del ojo izquierdo por sospecha de glaucoma secundario al uso crónico de dexametasona.

El diagnóstico final fue queratitis por rosácea, catarata y glaucoma. Se le aplicó tratamiento antibiótico con tetraciclinas, glucopéptidos y corticoesteroide (acetato de fluorometolona), y se suspendió la dexametasona.

La paciente no presentó mejoría, por lo que regresó a consulta refiriendo prurito en el ojo derecho. En el examen de agudeza visual, esta era del nivel de cuenta dedos a 2 m en ambos ojos, y la presión ocular en el ojo derecho era de 12 mm Hg y, en el izquierdo, de 14 mm Hg. En la biomicroscopía del ojo derecho, se observó eritema leve en el borde palpebral, secreción oleosa entre las pestañas, secreción blanquecina e hiperemia, córnea opalescente con líneas de Hudson-Stahli, irrigación profunda, iris con sinequias posteriores y catarata, en tanto que en el izquierdo se apreció eritema, secreción oleosa, borde palpebral engrosado, hiperemia simple, córnea con depósitos de lípidos, iris sin alteraciones, pupila reactiva y catarata. En el examen del fondo del ojo izquierdo se encontró una excavación del nervio óptico de 0,7.

La paciente recibió trasplante de córnea debido a la degeneración lipídica por rosácea ocular en el ojo derecho. Tres meses después de la cirugía, se produjo un rechazo agudo del trasplante con hiperemia mixta, botón corneal con edema y estrías en la membrana de Descemet. Se le administró tratamiento para el rechazo con esteroides tópicos y sistémicos sin obtener mejoría, por lo que se diagnosticó rechazo del trasplante de córnea. La paciente no se consideró candidata para un nuevo trasplante debido al mal pronóstico y se le recetó únicamente el tratamiento con lubricante en gel en el ojo derecho. El ojo izquierdo conservó el eje visual transparente, con opacidades periféricas. La catarata de este ojo era avanzada y requirió manejo quirúrgico, aunque con un pronóstico incierto.

## Discusión

Se describe la presentación, la evolución clínica y el manejo de la rosácea ocular en tres casos con características personales disímiles, pero con el factor común de la falta de sospecha diagnóstica inicial y de manifestaciones inespecíficas referidas por los pacientes, congruentes con los datos registrados en sus historias clínicas.

Si bien la rosácea es una alteración cutánea crónica que causa morbilidad ocular, la falta de sospecha diagnóstica puede afectar el pronóstico de los pacientes si la enfermedad no se diagnostica y se trata apropiadamente. El diagnóstico de la rosácea ocular es meramente clínico, sobre todo con el antecedente de rosácea cutánea, como se observó en el caso 1 del presente reporte. Sin embargo, hasta en el 20 % de los casos también puede presentarse con manifestaciones primarias y sin que haya antecedentes de enfermedades cutáneas, como ocurrió en los casos 2 y 3, en los que las manifestaciones cutáneas visibles no habían sido diagnosticadas de manera precisa ni oportuna ^6^^,^^7^. Por lo general, esta enfermedad afecta las glándulas de Meibomio y se ha reportado en el 10 % de los adultos. Entre las limitaciones para el diagnóstico precoz está la ausencia de alerta frente a las implicaciones a largo plazo de los síntomas y signos sugestivos de la rosácea leve, pues al no haber suficiente correlación diagnóstica con su causa etiológica, estos pueden subvalorarse. Si no hay coordinación entre los dermatólogos y los oftalmólogos, es incluso más difícil el diagnóstico oportuno, por lo que la sospecha en la consulta dermatológica debería conllevar comunicación con los especialistas del área oftalmológica, y viceversa ^7^.

No se conoce con exactitud la causa etiológica de la rosácea; se sabe que es una enfermedad multifactorial en la que la genética y el ambiente interaccionan, la reacción inmunitaria innata y la adaptativa se encuentran alteradas, la piel no funciona de manera óptima como barrera y la regulación de la microcirculación facial es anormal. El curso clínico se caracteriza por exacerbaciones y remisiones. Las primeras pueden desencadenarse por la exposición al sol y al calor, el tabaquismo y la ingestión de bebidas alcohólicas y alimentos picantes o condimentados. Además, la rosácea se ha asociado con enfermedades concurrentes como las infecciones por *Helicobacter pylori* o *Demodex folliculorum*, la enfermedad celiaca o la inflamatoria intestinal, entre otras. Algunos de los factores asociados descritos en la literatura científica se presentan en el cuadro 1 ^8^^-^^10^.

**Cuadro 1:** Factores asociados con el desarrollo y la aparición de la rosácea

**Agentes tópicos**	Cosméticos, retinoides, corticoesteroides
**Drogas**	Tabaco, niacina, nitroglicerina
**Alimentos**	Picante, chocolate, lácteos
**Factores emocionales**	Cambios de clima o de temperatura
**Miscelánea**	Ejercicio, cambios hormonales, cafeína, radiación

Los motivos de consulta más frecuentemente asociados con el diagnóstico de la rosácea ocular son la sensación de cuerpo extraño y la inflamación palpebral crónica ^6^, las cuales pueden estar acompañadas de signos primarios de la piel (telangiectasias, pústulas y pápulas de distribución simétrica) o manifestarse años después de la rosácea dermatológica ^2^.

Dados los síntomas oftalmológicos y dermatológicos que pueden sugerir la rosácea ocular, vale la pena mencionar las manifestaciones clínicas más comunes como párpados engrosados, secreción espesa de las glándulas de Meibomio (como pasta de dientes), conjuntiva hiperémica, fotofobia, irrigación irregular, córnea opalescente y secreciones oleosas en las pestañas, las cuales se pueden acompañar de cefalea no especificada. Por ser estos signos inespecíficos, es de suma importancia contar con historias clínicas y exploraciones físicas completas para un diagnóstico acertado y oportuno, lo que mejora el pronóstico del paciente ^8^.

Los pacientes aquí informados acudieron al Servicio de Oftalmología por presentar opacidades que disminuían su agudeza visual, por la sensación de cuerpo extraño y el engrosamiento de los párpados, síntomas que pueden considerarse los más comunes motivos de consulta. Cuando tres o más de estos síntomas coexisten, es dable la sospecha diagnóstica, sin dejar de lado los síntomas dermatológicos primarios que aparecen en la enfermedad, por lo que se sugiere hacer un examen físico completo y detallado, con énfasis en los aspectos dermatológicos y oculares.

En estudios recientes, se ha comprobado que el análisis de glucómica en las lágrimas y en la saliva de los pacientes con rosácea es un método diagnóstico que determina los biomarcadores específicos de la enfermedad. Sin embargo, actualmente no existe una prueba de diagnóstico confiable que permita detectar simultáneamente la afección dermatológica y la ocular ^8^.

En el curso de la enfermedad, pueden presentarse complicaciones como úlceras y perforación corneal, secuelas de la condición crónica que, incluso, llegan a confundirse con otras infecciones, lo que altera el tratamiento médico. Por lo general, tales complicaciones obligan a considerar opciones quirúrgicas como la queratoplastia lamelar profunda, la queratoplastia penetrante o, incluso, el trasplante total de córnea. Sin embargo, la evolución de estos pacientes no suele ser favorable y hasta en el 50 % de los casos se ha reportado la falla de los injertos cuando hay rosácea ^11^.

Dado que la córnea es un tejido avascular que, por lo general, presenta bajas tasas de rechazo al ser trasplantada, la principal causa de falla o fracaso del trasplante es el rechazo. El tratamiento del rechazo agudo del trasplante de córnea es con esteroides (inmunosupresores) tópicos, aunque en los trasplantes de alto riesgo puede ser necesaria la inmunosupresión sistémica. El marcador de pronóstico de las queratoplastias es el estado previo del ojo receptor, por lo tanto, su éxito depende de la causa del deterioro de la córnea. Se han propuesto cuatro pronósticos probables que se presentan en el cuadro 2 ^12^.

**Cuadro 2:** Pronóstico esperado en los pacientes con diagnóstico de rosácea ocular

Pronóstico	Estado de la córnea (receptor)	Fracaso quirúrgico (%)
Excelente	Córnea avascular más lesiones en la parte central	10
Bueno	Córnea avascular o leve irrigación más lesión corneal en la parte periférica	20
Regular	Córnea engrosada o con perforación o infección o inflamación activa	50
Malo	Hallazgos presentes: importante resequedad del ojo, isquemia conjuntival, cámara anterior plana y córnea irrigada	>50

La rosácea ocular suele asociarse con la queratitis, las perforaciones o las ulceraciones corneales. En los casos presentados el pronóstico era regular, con el 50 % de probabilidades de rechazo, razón por la cual podría considerarse de mal pronóstico. Puede concluirse que el trasplante de córnea en pacientes con esta condición depende, en parte, de la gravedad y la extensión de las lesiones de la córnea, aunque el diagnóstico temprano podría incrementar el porcentaje del éxito quirúrgico.

Los reportes consultados subrayan la importancia del pronto diagnóstico y el tratamiento del paciente con rosácea ocular, y de la consulta interdisciplinaria de oftalmólogos y dermatólogos para lograr un examen físico más integral y no tan enfocado en una sola especialidad; siempre se ha de favorecer la práctica clínica basada en la evidencia y en una adecuada coordinación de los equipos de salud. Se ha evidenciado que, cuanto más avanzada esté una enfermedad en el momento del diagnóstico, peor es el pronóstico, por lo que deben sumarse esfuerzos para evitarlo y facilitar el diagnóstico clínico y, en el futuro, incluso aquel basado en pruebas de laboratorio.

## Conclusión

Con base en los casos presentados, puede concluirse que en los pacientes de oftalmología con síntomas como disminución de agudeza visual asociada a episodios de ojo rojo recurrente, presencia de neoangiogénesis (*vascularization*) u opacidades corneales, y cambios característicos en los párpados, como engrosamiento palpebral, debe buscarse o indagarse intencionalmente sobre la presencia de compromiso dermatológico y antecedentes de rosácea. A pesar de presentarse con manifestaciones inespecíficas, la sospecha diagnóstica oportuna de la enfermedad puede ofrecer una rehabilitación más completa al paciente, ya que la detección extemporánea se acompañó en los casos reportados de cuadros clínicos graves, daño ocular extenso, intervenciones complejas y un pronóstico sombrío para la función visual y para el ojo.
